# The Regulatory Effect of SIRT1 on Extracellular Microenvironment Remodeling

**DOI:** 10.7150/ijbs.52619

**Published:** 2021-01-01

**Authors:** Zhuo Wang, Wendong Guo, Fei Yi, Tingting Zhou, Xiaoman Li, Yanling Feng, Qiqiang Guo, Hongde Xu, Xiaoyu Song, Liu Cao

**Affiliations:** College of Basic Medical Science, Institute of Translational Medicine, Key Laboratory of Medical Cell Biology, Ministry of Education, Key Laboratory of Liaoning Province, China Medical University, Shenyang, Liaoning Province, P.R. China, 110122.

**Keywords:** SIRT1, microenvironment remodeling, cell secretion, endocrine, inflammation, tumorigenesis

## Abstract

The sirtuins family is well known by its unique nicotinamide adenine dinucleotide (NAD^+^)-dependent deacetylase function. The most-investigated member of the family, Sirtuin 1 (SIRT1), accounts for deacetylating a broad range of transcription factors and coregulators, such as p53, the Forkhead box O (FOXO), and so on. It serves as a pivotal regulator in various intracellular biological processes, including energy metabolism, DNA damage response, genome stability maintenance and tumorigenesis. Although the most attention has been focused on its intracellular functions, the regulatory effect on extracellular microenvironment remodeling of SIRT1 has been recognized by researchers recently. SIRT1 can regulate cell secretion process and participate in glucose metabolism, neuroendocrine function, inflammation and tumorigenesis. Here, we review the advances in the understanding of SIRT1 on remodeling the extracellular microenvironment, which may provide new ideas for pathogenesis investigation and guidance for clinical treatment.

## Introduction

Extracellular microenvironment has been considered as a crucial element that influences cellular proliferation and metabolism 1, 2. It mediates the communication and interaction between neighboring cells, different tissue cells, and even distinct organs for the insurance of normal functions 3, 4. Meanwhile, the extracellular microenvironment can be reformed by intracellular proteins through regulating the processes and cargos of cell secretion 5. As the significant nicotinamide adenine dinucleotide-dependent protein deacetylase, sirtuin 1 (SIRT1) is extensively involved in various cellular processes and metabolism 6, 7. Our previous work revealed that SIRT1 played important roles in genome stability maintenance 8, DNA damage response 9 and autophagy 10. We further realize that SIRT1 not only affects intracellular homeostasis, but also participates in the extracellular microenvironment remodeling. In this study, we review the related literatures focusing on SIRT1 function as a cell secretion regulator, hoping to find new ways and targets for future research and clinical treatment.

## SIRT1 modulates glucose metabolism through cell secretion

**Figure 1:**
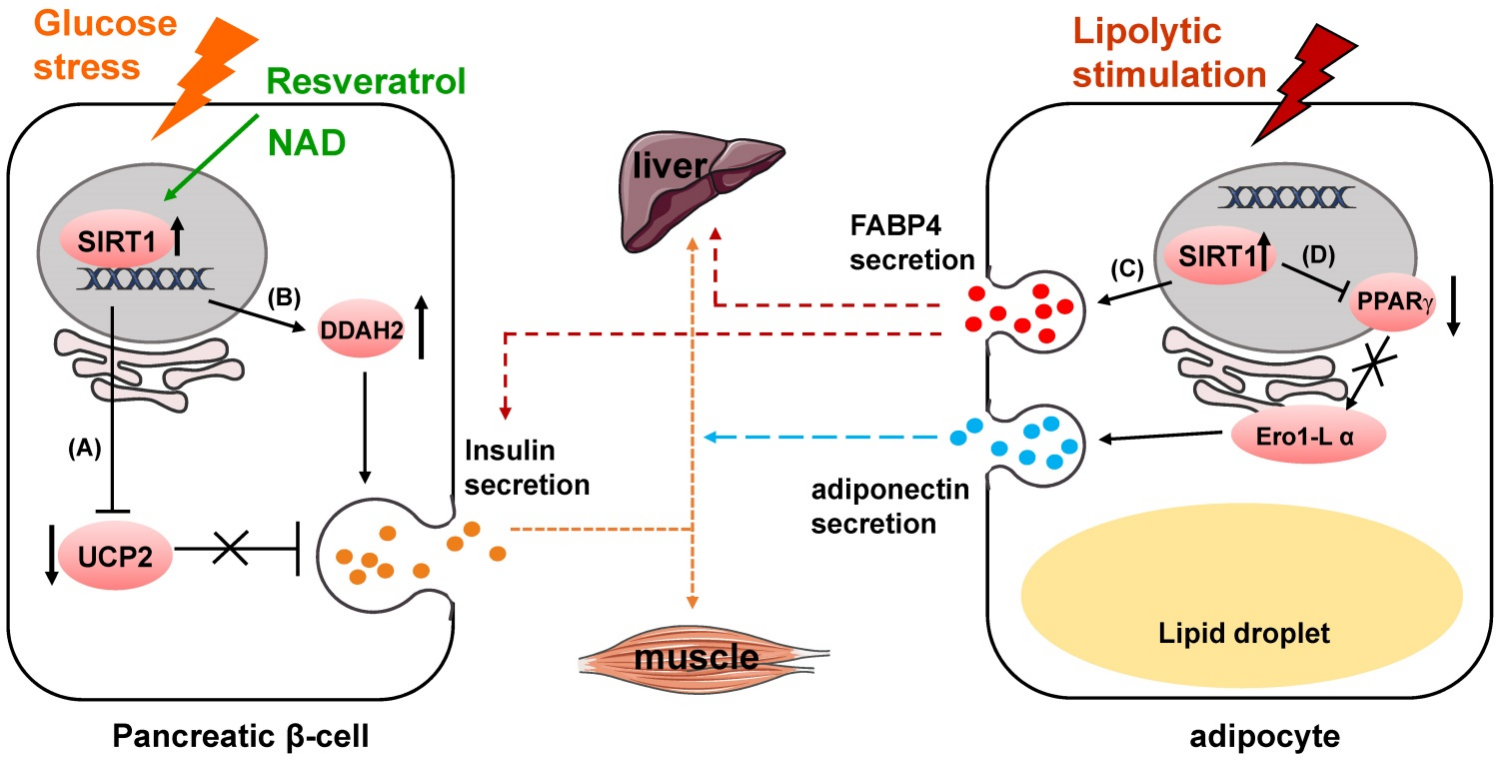
SIRT1 modulates glucose metabolism through regulating cell secretion. (A) SIRT1 suppresses UCP2 expression to upregulate insulin secretion. (B) SIRT1 enhances insulin secretion through DDAH2/secretagogin pathway. (C) SIRT1 increases FABP4 secretion to regulate hepatic glucose production and glucose-stimulated insulin secretion. (D) SIRT1 inhibits PPARγ activity to downregulate Ero1-L α expression, and suppresses adiponectin secretion.

SIRT1 has got long-term attention and well known for playing a pivotal role in glucose homeostasis and Type 2 Diabetes 11, 12. One of its functions is to regulate insulin secretion. In pancreatic beta cells, SIRT1 promotes insulin secretion in response to glucose stress by suppressing the expression of uncoupling protein 2 (UCP2) 13-15 (Fig. 1A). As a negative regulator of insulin, increased expression of UCP2 in the pancreatic β-cells results in the decrease of glucose-stimulated insulin secretion (GSIS) leading to pancreatic β-cell dysfunction and development of type-II diabetes 16, 17. UCP2 is shown to regulate glucose metabolism and insulin secretion through many biological activities, including reducing NADH levels, decreasing ATP production, weakening mitochondrial membrane potential and suppressing generation of superoxide 18-20. SIRT1 can also enhance insulin secretion of pancreatic β-cells through the hydrolase dimethylarginine dimethylaminohydrolase 2 (DDAH2)/secretagogin pathway 21 (Fig. 1B). SIRT1 boosts DDAH2 expression on the transcriptional level by activating promoter of 5' deletion constructs. The overexpression of DDAH2 induces the upregulation of secretagogin, an EF-hand Ca2^+^-binding protein which is involved in vesicle secretion 22. Besides, DDAH2 was reported to have directly interaction with secretagogin 23.

Furthermore, SIRT1 itself can be regulated by in the process of insulin secretion 24. MicroRNA mir-9 targets and reduces SIRT1 protein level during glucose-dependent insulin secretion. Some protein and compounds can also influence insulin secretion through regulating SIRT1 activity. For instance, Wallerian degeneration slow (WldS), a fusion protein with NAD biosynthesis activity, can increase NAD level, which leads to the enhanced SIRT1 activity to downregulate UCP2 25, 26. Resveratrol, currently a most potent natural compound SIRT1 activator, enhances insulin secretion in human islets in response to both glucose and high fat diet 27, 28. Additionally, resveratrol improves insulin induced NO secretion partly through activating SIRT1 in endothelial cells 29. This effect might be related to improvement of endothelial cell function in animal models and in humans 30-32.

In mouse models, β-cell specific SIRT1-overexpression transgenic mice have presented enhanced glucose-stimulated insulin secretion and improved glucose tolerance at age of 3 and 8 months 13. However, the phenotype completely vanishes when the transgenic mice reach 18 to 24 months 33. Meanwhile, in these mouse models, decreased SIRT1 expression impairs glucose sensing as well as insulin secretion 34, 35.

In mature adipocytes, SIRT1 negatively regulates adiponectin secretion through inhibiting peroxisome proliferator activated receptor γ (PPARγ) activity 36 (Fig. 1D). Suppression of SIRT1 or activation of PPARγ upregulates the protein level of endoplasmic reticulum oxidoreductase 1 α (Ero1-L α) and stimulates secretion of high-molecular-weight adiponectin. The secreted complexes of adiponectin was reported to sensitize liver and muscle cells to insulin in response to various metabolic states 37, 38. Secretion of fatty acid binding protein 4 (FABP4), a lipid carrier protein, from white adipose tissue is dependent on SIRT1 in response to lipolytic stimulation 39. The secretion process also requires early components of autophagy such as beclin-1. The secreted FABP4 in circulating system is a signal molecule transmitted from adipose tissue to liver to augment the production of hepatic glucose 40 (Fig. 1C). Circulating FABP4 level is positively correlated with glucose-stimulated insulin secretion 41.

## SIRT1 regulates lipid metabolism through cell secretion

Various researches have proved that SIRT1 plays a role in regulating lipid metabolism 42. Among those reports, SIRT1 was found to be a protective factor in the development of atherosclerosis 43-45. SIRT1 inhibits secretion of thrombosis promoting factors, von Willebrand factor (vWF) and P-selectin, from vascular endothelial cells, thus preventing thrombosis formation 46. This effect is probably relevant to the regulation of autophagy through SIRT1/FOXO1 pathway. According to Miranda's work, SIRT1 activation reduces hepatic secretion of a serine protease, proprotein convertase subtilisin/kexin type 9 (PCSK9). The secreted mediates lysosomal degradation of hepatic low-density lipoprotein receptor (LDLR) and prevents its internalizing recycle to cell surface 47. PCSK9 accumulation increases LDLR protein degradation and then enhances LDL-cholesterol plasma clearance, leading to decreased plaque formation 48, 49. These results are consistent with reduced levels of blood cholesterol and adipokines in SIRT1 transgenic mice 50-52.

## SIRT1 contributes to neuroendocrine secretion

**Figure 2:**
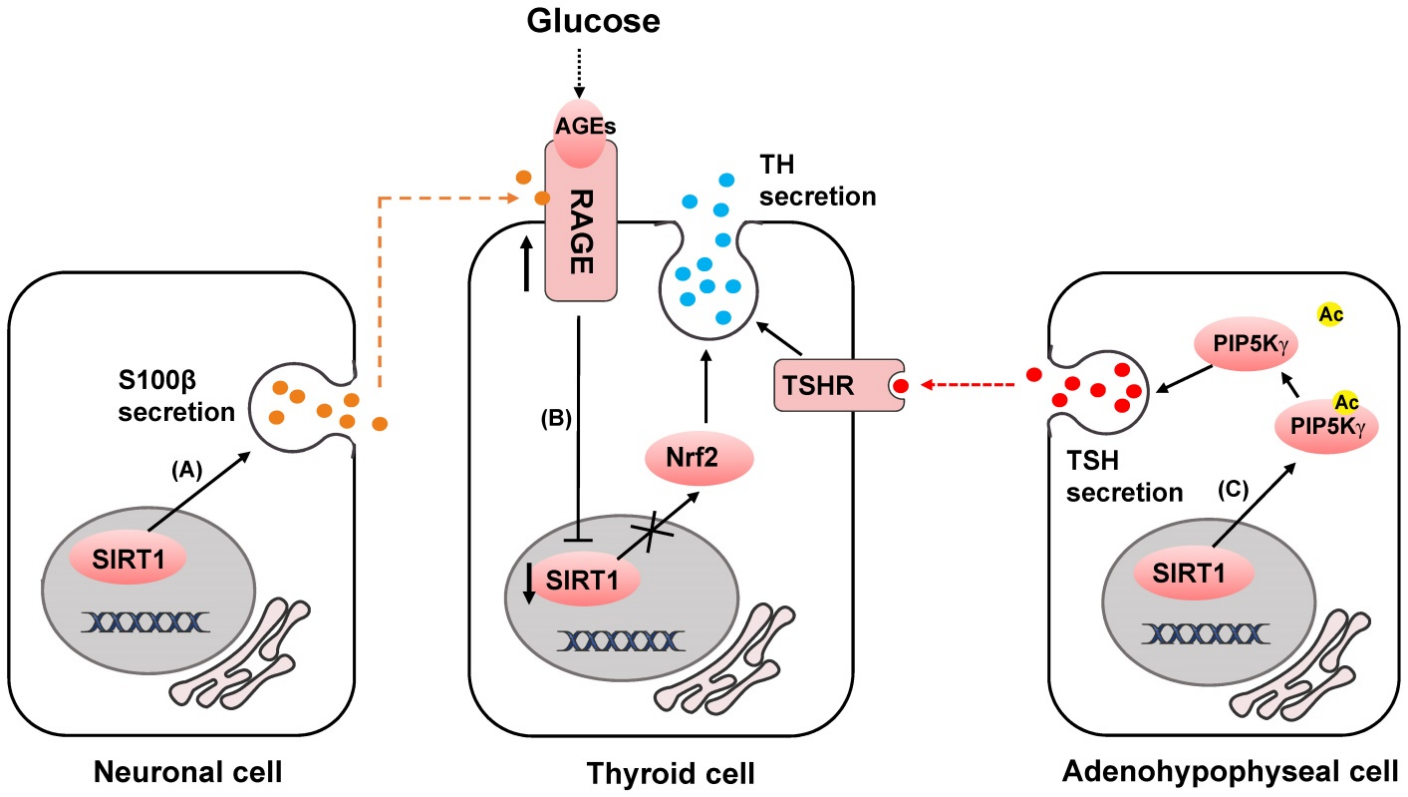
SIRT1 regulates neuroendocrine secretion. (A) SIRT1 upregulates S100β release. (B) AGEs bind to RAGE and inactivates SIRT1/NRF2 pathway, and decreases TH secretion. (C) SIRT1 deacetylates PIP5Kγ to increase TSH release, and stimulates TH secretion.

In clinical work, Diabetes mellitus is often found to be coexisted with hypothyroidism 53. This might be partially related to the upregulation of advanced glycation end products (AGEs), high glucose induce advanced glycation end products receptor (RAGE) and the inactivation of SIRT1/nuclear factor erythroid 2-related factor (NRF2) pathway 54-56 (Fig. 2B). High glucose and AGEs induce upregulation of RAGE, downregulation of SIRT1 and NRF2, and decrease of proteins related to thyroid hormone (TH) secretion, thus finally resulting in decreased TH secretion and circulating TH deficiency (Fig. 2C). Sayaka et al reveals that in pituitary gland, SIRT1 multiples exocytosis of thyroid stimulating hormone (TSH) containing granules by deacetylating phosphatidylinositol-4-phosphate 5-kinase γ (PIP5Kγ) 57, which mainly mediates the large dense-core vesicle fusion 58 (Fig. 2C). Consistently, high acetylated PIP5Kγ and decreased thyroid hormone in the plasma were observed in SIRT1 knock out mice 57, 59. Hormone disorder displayed in neuron-specific SIRT1 knock out mice showed that more work still needs to be done to address how SIRT1 modulates neural cell secretion and somatotropic signaling 60.

In previous studies, SIRT1 was found to be predominantly expressed in neurons and highly involved in neurodegenerative diseases 61, 62. SIRT1 is revealed to be neuroprotective in amyloid-β-induced ROS production, DNA damage and oxidative modifications 63, 64. Qin et al identify in C6 rat glioma cells that enhanced expression of SIRT1 can upregulate the release of a calcium-binding protein S100β 65. The extracellular S100β functions as cytokines with both neurotrophic and neurotoxic effects and affects the activity of several cell types, such as neurons 66-68, astrocytes 69, 70, and microglia 71, 72, through the surface receptor RAGE 73-76 (Fig. 2A).

Moreover, SIRT1 positively affects the NAD biosynthesis in the hypothalamus by distinctly regulating the release of Nicotinamide phosphoribosyl transferase (NAMPT) in adipose tissue. In this process, SIRT1 predisposes NAMPT to be secreted to the extracellular enzyme pool by deacetylating it at lysine 53 77.

## SIRT1 and inflammatory microenvironment

**Figure 3:**
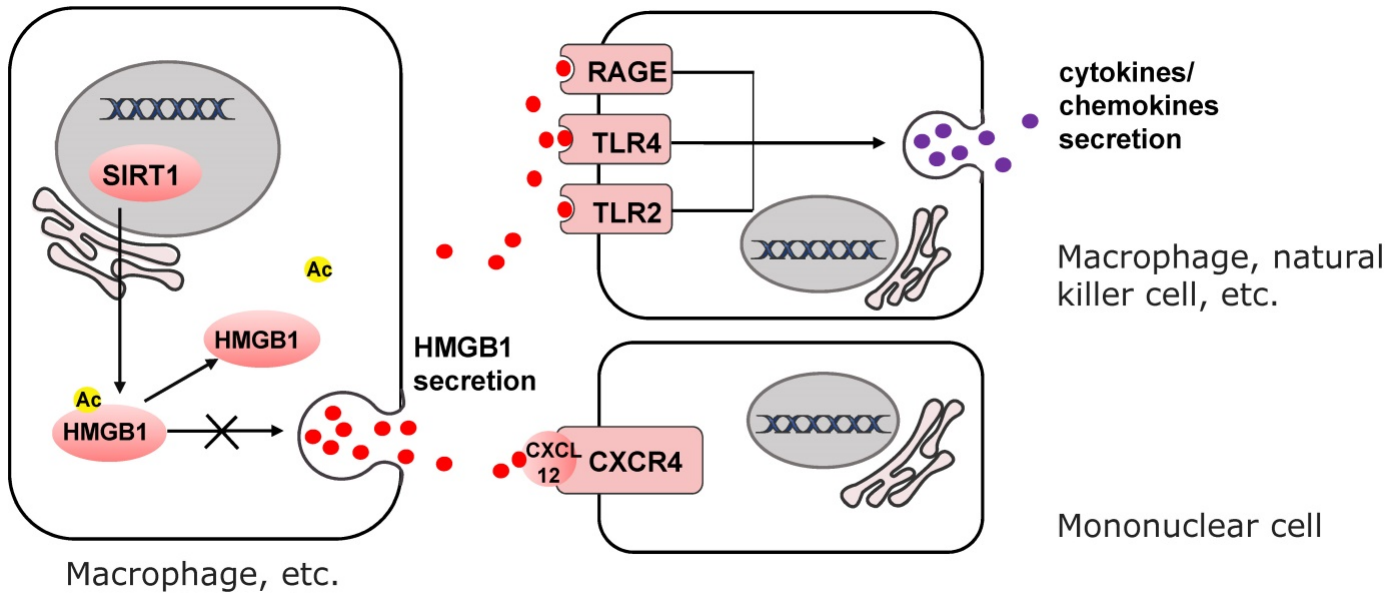
SIRT1 mediates inflammatory microenvironment remodeling by deacetylating HMGB1 to reduce its secretion. Released HMGB1 forms complex with CXCL12 and CXCR4 to induce the recruitment of mononuclear cells. HMGB1 also binds to TLR2, TLR4 and RAGE to mediate cytokines or chemokines secretion of immune cells.

High mobility group box 1 (HMGB1) was identified as a structural protein of chromatin which functioning in transcription 78, 79. But when secreted into the extracellular microenvironment, it induces acute and chronic inflammation as a proinflammatory cytokine 80-83. HMGB1 mediates the recruitment of mononuclear cells 84, the release of cytokines or chemokines 85-87, and the activation of effector T cells and suppression of regulatory T cells^88^, ^89^ (Fig. 3). The exocytosis of HMGB1 is highly dependent on its level of acetylation 90. SIRT1 attenuates the exocytosis of HMGB1 through deacetylation 91-93. The secretion of HMGB1 into the extracellular microenvironment can be downregulated by upregulating the activity or expression of SIRT1 91, 94. This SIRT1-mediated mechanism has been described in Zeng's research and can prevent non-alcoholic fatty liver disease induced by high fat diet 95. In response to the stimulation of H_2_O_2_, suppression of SIRT1 leads to the upregulation of HMGB1 released from hepatocytes 96. Intriguingly, secretion of HMGB1 from kidney cells increases in early stage of hemorrhagic shock by downregulation of SIRT1 expression level 97. In turn, downregulation of circulating HMGB1 by SIRT1 protects liver from ischemic injury 98. This phenomenon portends that the secretion of HMGB1 changes in different organs and extracellular microenvironment dependent on SIRT1 activity.

## SIRT1 and tumor microenvironment

SIRT1 is proved to be highly involved in cancer because of its underlying functions in tumorigenesis 99-101, senescence 102, 103, immunity 104, 105 and inflammation 106. Chronic lymphocytic leukemia creates its suitable microenvironment for survival through the release of aforementioned protein, HMGB1 107. Besides, the extracellular S100β, which can be regulated by SIRT1, participates in the recruitment and activity modulation of monocytes in tumor microenvironment 65, 108, 109. A recent study exhibits that inhibition of SIRT1 by caveolin-1 in senescent fibroblasts promotes the secretion of interleukin 6 (IL-6) and stimulates tumor growth 110.

**Figure 4:**
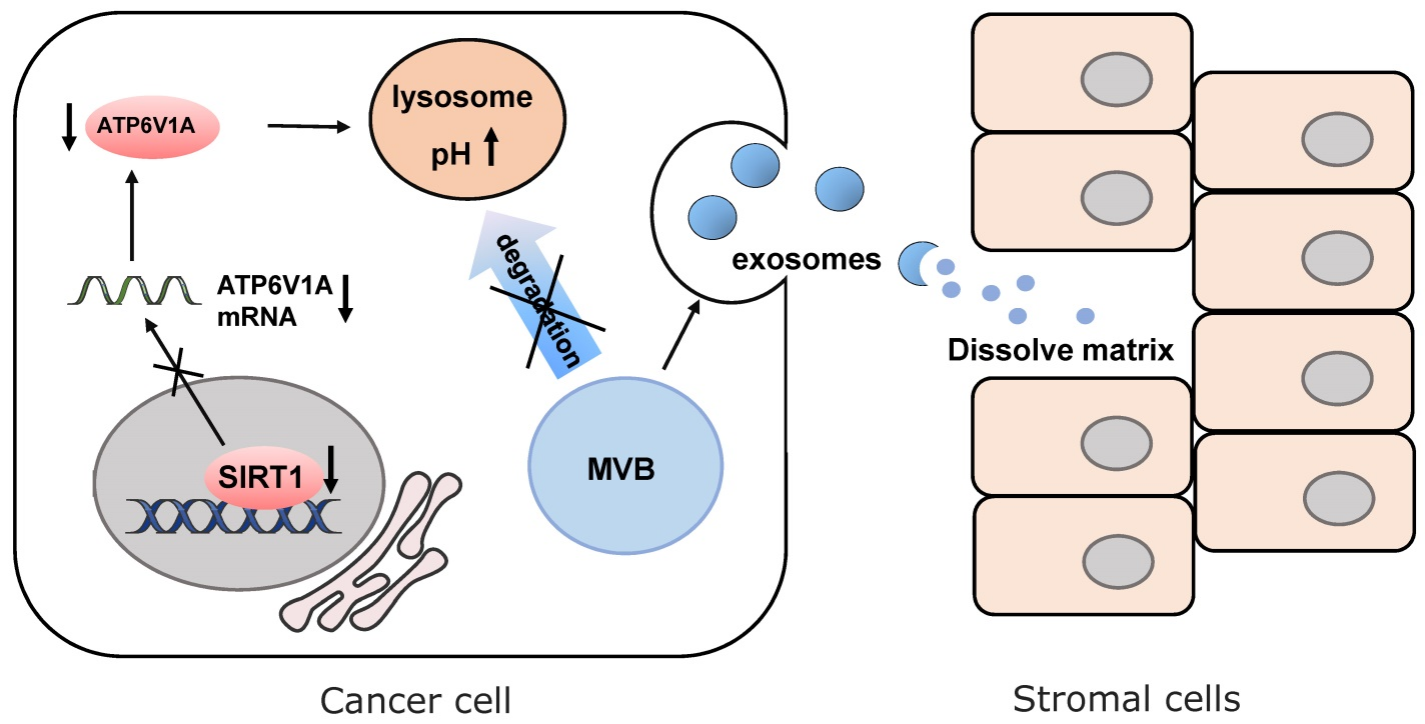
SIRT1 increases the expression of ATP6V1A to maintain pH level of lysosomes. Down-regulation of SIRT1 promotes MVBs formation and enhancing protein cargos released by exosomes to dissolve the cell matrix of tumor microenvironment.

The latest progress discovers that SIRT1 is responsible for the change of microenvironment by regulating the secretion of exosomes 111 (Fig. 4). In breast cancer, loss of SIRT1 enhances the secretion of pro-tumorigenic exosomes and promotes cancer invasion 112. In triple-negative breast cancer, down-regulating SIRT1 levels decreases the expression of ATPase H^+^ transporting V1 subunit A (ATP6V1A), a particular subunit of the vacuolar-type H^+^ ATPase (V-ATPase). It is responsible for acidification of lysosomes and degradation of protein. Disruption of the degradation process leads to the reduction of multi-vesicular bodies (MVBs) and the formation of larger MVBs. Finally, the imbalanced MVB formation promotes enhancing protein cargos released by exosomes 113, 114. The released cargos dissolve the extracellular matrix of normal cells adjacent to tumor cells and destroy para cancer tissue structures. This SIRT1-dependent mechanism enables cancer cells to create the suitable extracellular microenvironment for the expansion of themselves. In senescent stromal cells, SIRT1-loss also causes impairment of lysosomes acidification and protein degradation. That is why senescent cells presumably prefer to release small extracellular vesicles into the tumor microenvironment, which enhances the aggressiveness and drug resistance of recipient cancer cells mediated by ATP binding cassette subfamily B member 4 (ABCB4) 115.

## Conclusions

Accumulating evidences suggest that SIRT1 has wide effects on regulating extracellular microenvironment through cell secretion. Changes of expression or activity of SIRT1 can result in functional variations of the neuroendocrine system, inflammatory and tumor microenvironment through driving proteins, cargos in exocytosis vesicles and exosomes secreting into extracellular microenvironment. The released proteins functions as enzymes, cytokines, neuroendocrine factors and ligands of cell surface receptors. These molecules regulate downstream target cells bringing about different effects. These variations are involved in glucose metabolism, TH/TSH secretion, lipid metabolism, inflammation in thrombosis, tumorigenesis and metastasis.

We should give more concern to the extracellular microenvironment remodeling function of SIRT1 in scientific researches and clinical treatment. Further research on new drug design may focus on its regulatory effect of extracellular mechanisms, targeting SIRT1 activity or the secreted downstream proteins 116, 117.

It is worth noting that the SIRT1 dependent HMGB1 secretion in kidney and liver shows different alterations in ischemic injury 97, 98. This prompts that SIRT1-dependent extracellular microenvironment regulation may be variant in different organs and cause opposite results. Thus, in using drugs targeted for SIRT1 in clinical treatment or assessing the medical effects, the specific extracellular effects should be taken into consideration.

## Abbreviations

ABCB4: ATP binding cassette subfamily B member 4
AGEs: advanced glycation end products
ATP6V1A: ATPase H+ transporting V1 subunit A
CXCL12: C-X-C motif chemokine ligand 12
CXCR4: C-X-C motif chemokine receptor 4
DDAH2: dimethylarginine dimethylaminohydrolase 2
Ero1-L α: endoplasmic reticulum oxidoreductase 1 alpha
FABP4: fatty acid binding protein 4
FOXO: Forkhead box O
GSIS: glucose-stimulated insulin secretion
HMGB1: High mobility group box 1
IL-6: interleukin 6
LDLR: low-density lipoprotein receptor
LPS: lipopolysaccharide
MVBs: multi-vesicular bodies
NAD: nicotinamide adenine dinucleotide
NAMPT: nicotinamide phosphoribosyl transferase
NRF2: nuclear factor erythroid 2-related factor
PCSK9: proprotein convertase subtilisin/kexin type 9
PIP5Kγ: phosphatidylinositol-4-phosphate 5-kinase γ
PPARγ: peroxisome proliferator activated receptor γ
RAGE: advanced glycation end products receptor
SIRT1: sirtuin 1
TH: thyroid hormone
TLR2: toll like receptor 2
TLR4: toll like receptor 4
TSH: thyroid stimulating hormone
UCP2: uncoupling protein 2
V-ATPase: vacuolar-type H^+^ ATPase
vWF: von Willebrand factor
WldS: Wallerian degeneration slow
